# Reducing In-Stent Restenosis: Therapeutic Manipulation of miRNA in Vascular Remodeling and Inflammation

**DOI:** 10.1016/j.jacc.2015.03.549

**Published:** 2015-06-02

**Authors:** Robert A. McDonald, Crawford A. Halliday, Ashley M. Miller, Louise A. Diver, Rachel S. Dakin, Jennifer Montgomery, Martin W. McBride, Simon Kennedy, John D. McClure, Keith E. Robertson, Gillian Douglas, Keith M. Channon, Keith G. Oldroyd, Andrew H. Baker

**Affiliations:** ∗ Institute of Cardiovascular and Medical Sciences, College of Medical, Veterinary and Life Sciences, University of Glasgow, Glasgow, Scotland; † West of Scotland Regional Heart & Lung Centre, Golden Jubilee National Hospital, Clydebank, Scotland; ‡ Department of Cardiovascular Medicine, University of Oxford, John Radcliffe Hospital, Oxford, United Kingdom

**Keywords:** late stent thrombosis, miRNA stem loop, neointima, smooth muscle cell, BMDM, bone marrow–derived macrophages, BMS, bare-metal stent(s), CD, cluster of differentiation, DES, drug-eluting stent(s), IL, interleukin, ISR, in-stent restenosis, KO, knockout, LPS, lipopolysaccharide, miRNA, micro–ribonucleic acid, PDCD4, programmed cell death protein 4, PDGF, platelet-derived growth factor, PPAR, peroxisome proliferator–activated receptor, RNA, ribonucleic acid, SMC, smooth muscle cell, WT, wild-type

## Abstract

**Background:**

Drug-eluting stents reduce the incidence of in-stent restenosis, but they result in delayed arterial healing and are associated with a chronic inflammatory response and hypersensitivity reactions. Identifying novel interventions to enhance wound healing and reduce the inflammatory response may improve long-term clinical outcomes. Micro–ribonucleic acids (miRNAs) are noncoding small ribonucleic acids that play a prominent role in the initiation and resolution of inflammation after vascular injury.

**Objectives:**

This study sought to identify miRNA regulation and function after implantation of bare-metal and drug-eluting stents.

**Methods:**

Pig, mouse, and in vitro models were used to investigate the role of miRNA in in-stent restenosis.

**Results:**

We documented a subset of inflammatory miRNAs activated after stenting in pigs, including the miR-21 stem loop miRNAs. Genetic ablation of the miR-21 stem loop attenuated neointimal formation in mice post-stenting. This occurred via enhanced levels of anti-inflammatory M2 macrophages coupled with an impaired sensitivity of smooth muscle cells to respond to vascular activation.

**Conclusions:**

MiR-21 plays a prominent role in promoting vascular inflammation and remodeling after stent injury. MiRNA-mediated modulation of the inflammatory response post-stenting may have therapeutic potential to accelerate wound healing and enhance the clinical efficacy of stenting.

Coronary stenting has almost universally superseded the use of balloon angioplasty alone for the percutaneous treatment of coronary heart disease. Stenting solves the major problems of balloon angioplasty, including acute elastic recoil, occlusive dissection, and the need for repeat revascularization due to restenosis (1). Stenting is superior to balloon angioplasty alone in the setting of both stable and unstable coronary artery disease 2, 3. However, the vascular injury caused by stent implantation provokes neointimal hyperplasia due to aberrant vascular smooth muscle cell (SMC) proliferation and migration (4). The resulting encroachment on the vessel lumen may be sufficient to cause in-stent restenosis (ISR), recurrent ischemia, and a need for repeat revascularization in up to 20% of patients treated with bare-metal stents (BMS) at 1 year. The development of metallic drug-eluting stents (DES) coated with an antiproliferative drug has substantially reduced ISR (5) but is associated with a significantly greater incidence of late stent thrombosis compared with BMS due to delayed arterial healing 6, 7. Several clinical trials are currently evaluating fully bioresorbable nonmetallic DES, but early reports suggest that they may have higher rates of incomplete strut apposition and strut fracture 8, 9. Collectively, these findings highlight the need to further improve our understanding of the events that control vascular healing responses with both BMS and DES.

Noncoding ribonucleic acids (RNAs) play a pivotal role in many physiological and homeostatic processes 10, 11. The best characterized are short, highly conserved RNA molecules called microRNAs (miRNAs), which mediate messenger RNA silencing through translational degradation or repression after complementary base pairing (12). More than 1,000 miRNAs are thought to regulate ∼30% of all protein-coding messenger RNA (13). Thus, a single miRNA may have ≥1 messenger RNA targets at different points within multiple biological pathways to mediate a disease phenotype 14, 15. In the setting of vascular injury, miRNAs are involved in inflammatory cell recruitment and activation and dedifferentiation of SMCs, key processes that drive the vessel response to injury. However, there has been no systematic analysis of miRNA regulation post-stent deployment.

The goal of the present study, therefore, was to define the expression pattern and function of miRNAs after stenting with BMS and DES to identify miRNAs with the potential to modulate vascular response to injury.

## Methods

Detailed methods are available in the Online Appendix. In brief, male Landrace pigs (19 to 26 kg) were pre-dosed orally with aspirin and clopidogrel 24 h before surgery, and they were maintained on this dual antiplatelet therapy throughout the study to reduce the risk of in-stent thrombosis. Vascular access was obtained by femoral artery cutdown and the insertion of a 6-F transradial sheath (Arrow International, Reading, Pennsylvania). Coronary angiography was performed before the deployment of either BMS (Gazelle, Biosensors Europe SA, Morges, Switzerland) or Biolimus A9 eluting stents (BioMatrix Flex, Biosensors Europe SA) to achieve a target ratio of stent to artery diameter of 1.2:1. Animals were euthanized after 7 or 28 days.

In the murine model, a stainless steel stent (5-cell, 2.5 mm × 0.8 mm; strut thickness 0.06 mm; Cambus Medical, Galway, Ireland) was crimped onto a 1.20-mm × 8-mm Mini-Trek balloon angioplasty catheter (Abbott Vascular, Abbott Park, Illinois) and deployed (10 atm for 30 s) into the thoracic aorta before engraftment into a recipient mouse. Mice were allowed to recover in heated chambers for 24 h and were returned to normal housing conditions, where they were maintained on aspirin-supplemented water and a normal chow diet for another 28 days before being killed. Murine aortas were harvested from male mice 8 to 12 weeks of age, and vascular SMCs were isolated and cultured. Cell migration was assessed via scratch assay. Bone marrow–derived macrophages (BMDM) were generated and bone marrow cells isolated from femurs and tibiae of wild-type (WT) and miR-21 knockout (KO) mice. Flow cytometry was performed by using a BD FACSCanto II or LSR II (BD Biosciences, San Jose, California).

Global profiling for miRNAs in the control unstented arteries and stented porcine coronary arteries was performed with miRNA quantification by using real-time polymerase chain reaction. For analysis, fold changes for each miRNA were normalized to U6 because this miRNA was the most suitable endogenous miRNA in porcine tissue. Data analysis was performed by using SDS software version 2.3 (Applied Biosystems, Carlsbad, California), and the baseline and threshold were automatically set. Data were normalized and then analyzed to identify miRNAs differentially expressed between the control (unstented) arteries and arteries subjected to stenting for 7 or 28 days. Data were analyzed by using DataAssist software version 3 (Applied Biosystems).

All data are mean ± SEM. Visual assessment was used to check for any lack of normality; because there was no evidence of this, 1-way analysis of variance followed by a Tukey multiple comparison test (for comparison of >2 groups) or Student *t* test (for comparison of 2 groups) were conducted. For all the quantitative polymerase chain reaction experiments, values are expressed as fold-change. All statistical analyses were conducted by using Prism version 4 (GraphPad Software, San Diego, California). The microRNA array data were analyzed in DataAssist software. Comparisons of in vitro SMC proliferation and migration were performed by using 2-way analysis of variance and Bonferroni post-hoc tests.

All animal procedures were performed in accordance with the United Kingdom Home Office Guidance on the operation of the Animals (Scientific Procedures) Act 1986 and institutional ethical approval.

## Results

**Figure 1:**
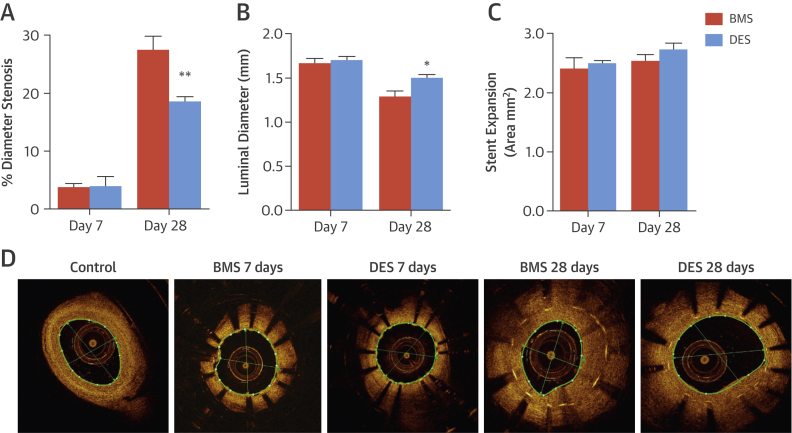
ISR Assessment: Porcine Stent Model Porcine coronary arteries were stented with bare-metal stents (BMS) or Biolimus A9 drug-eluting stents (DES). Measurements of in-stent restenosis (ISR), including **(A)** percent diameter stenosis, **(B)** luminal diameter, and **(C)** stent expansion, were analyzed at 7 and 28 days post-stent placement. **(D)** Representative optical coherence tomography images show control (unstented) arteries and arteries stented with BMS or DES for a period of 7 or 28 days. *p < 0.05; **p < 0.01 versus BMS day 28 (1-way analysis of variance).

The effects of BMS and DES on ISR were assessed in pig coronary arteries by using optical coherence tomography imaging (Figures 1A to 1D). The DES reduced neointima formation by 33% compared with BMS at 28 days (Figure 1A), leading to a significantly larger luminal area (Figure 1B) with no difference in stent expansion (Figure 1C).

**Figure 2:**
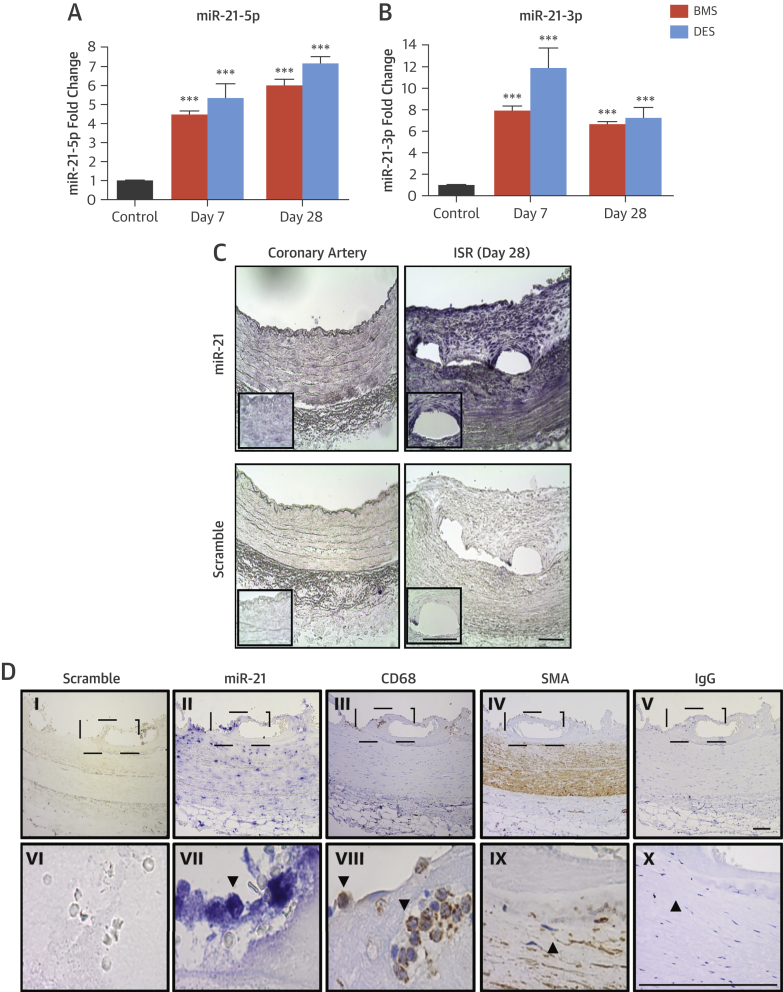
Regulation of miR-21-5p and -3p During ISR Relative-fold change in **(A)** miR-21-5p and **(B)** miR-21-3p is expressed in control arteries or arteries stented with a BMS or DES for 7 or 28 days. ***p < 0.001 versus control arteries (1-way analysis of variance). Data are normalized to expression of U6. **(C)** In situ hybridization for miR-21 and scrambled control in unstented porcine coronary arteries and in BMS-stented vessels with ISR at day 28 (representative images, n = 3). Areas under enhanced magnification correspond to the regions highlighted by the hatched rectangles. Scale bar = 100 μm. **(D)** In situ hybridization for miR-21 and scrambled control in stented human coronary arteries and immunohistochemistry for smooth muscle actin (SMA) and cluster of differentiation (CD) 68 for smooth muscle cells and macrophages, respectively. Panels VI through X are higher magnification images of the hatch regions in panels I through V. Scale bar = 100 μm. IgG = immunoglobulin G; other abbreviations as in Figure 1.

We sought to identify aberrantly expressed miRNAs. At 7 days post-stenting, 116 miRNAs were differentially regulated in the BMS group, with 23 miRNAs remaining dysregulated at 28 days. At 7 days, multiple miRNAs associated with inflammatory cell infiltration and activation were altered (Online Table 1, Online Figure 1). Of note, miR-21-3p was substantially up-regulated in both BMS- and DES-treated arteries at 7 days, suggesting that the miR-21 stem loop (i.e., both lead and “passenger” strands) may be important post-stenting. The expression profile of miR-21-3p and miR-21-5p were validated, and this revealed that miR-21-5p was significantly up-regulated in stented arteries at 7 days regardless of stent type and remained up-regulated at 28 days compared with unstented control arteries (Figure 2A). MiR-21-3p was also up-regulated at 7 and 28 days post-stenting in both groups (Figure 2B). We then identified the localization of expression of the miR-21 axis in the vessel wall after stenting, focusing on miR-21-5p (because the absolute levels of miR-21-3p are lower and below the detection threshold for in situ hybridization). In control coronary arteries, miR-21-5p was detected within the media (Figure 2C). In injured vessels 28 days post-stenting, increased signal intensity was observed in the medial layer and developing neointima, particularly around stent struts. To determine if this expression pattern is maintained in the clinical setting, in situ hybridization was performed in human coronary arteries 14 days post-stenting from tissue obtained from a heart transplant patient. In concordance with the staining pattern in porcine arteries, abundant miR-21 expression was observed in the developing neointima and infiltrating inflammatory cells (Figure 2D, Online Figure 2). Taken together, these findings suggest that miR-21-5p and miR-21-3p may be important in the development of post-stent pathophysiological responses to injury.

**Figure 3:**
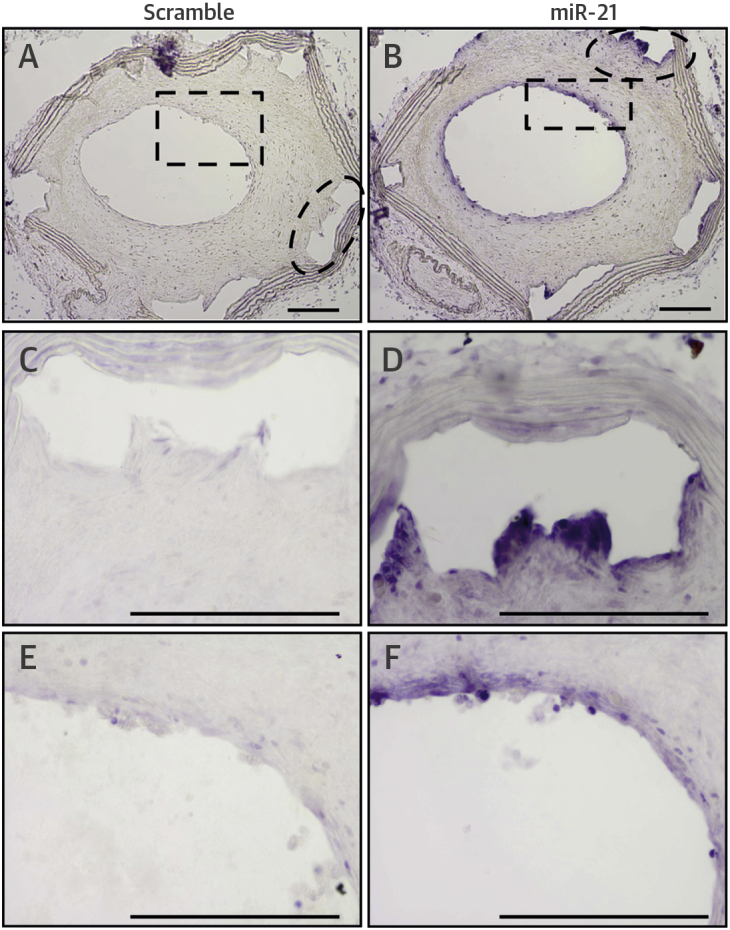
In Situ Hybridization for miR-21 Scramble probe at **(A)** low magnification and **(C and E**) areas under enhanced magnification corresponding to regions highlighted by the **hatched areas** are seen in murine stented grafts 28 days post-implantation, as are **(B)** MiR-21 probe and **(D and F)** areas under enhanced magnification corresponding to regions highlighted by the **hatched areas**. Scale bar = 100 μm.

**Figure 4:**
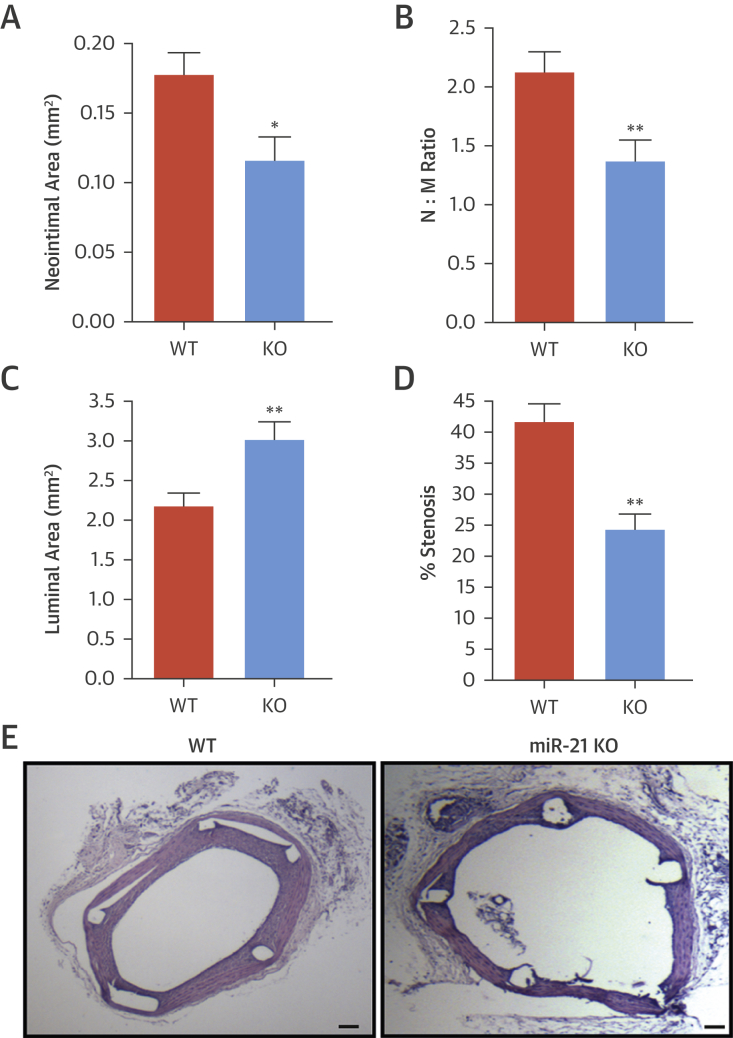
Effect of miR-21 Ablation on Neointimal Formation and ISR Morphometric analysis of stented aortic grafts of wild-type (WT) and miR-21 knockout (KO) mice 28 days after stent placement show **(A)** neointimal area, **(B)** neointimal/media (N/M) ratio, **(C)** luminal area, and **(D)** percent stenosis. *p < 0.05; **p < 0.01 versus WT mice. **(E)** Representative hematoxylin and eosin–stained sections from WT and miR-21 KO mice are seen 28 days post-stenting. Scale bar = 200 μm. Abbreviations as in Figure 1.

We used a murine interpositional graft model of stenting. In situ hybridization confirmed abundant miR-21-5p staining around the stent struts and developing neointima (Figure 3), consistent with the porcine data. Morphometric analysis found that miR-21 KO mice stent grafts had a reduced neointimal area, neointimal thickness, and neointima-to-medial ratio compared with WT controls (1.37 ± 0.18 [n = 8] vs. 2.11 ± 0.17 [n = 9]; p < 0.05) (Figures 4A and 4B, Online Table 2). Measurements of strut depth were concordant with greater strut depth in WT-stented grafts than in KO-stented grafts. A significant reduction in luminal area in WT mice compared with miR-21 KO mice was also observed (Figures 4C and 4D, Online Table 2). There were no apparent differences in luminal, media, or total vessel area between WT and miR-21 KO mice at baseline (before stent injury), although an increased sample size would be needed to confirm this observation (Online Table 3). Furthermore, the total vessel area did not differ significantly at 28 days (Figure 4E), and no differences were observed in unstented vessels at baseline (Online Table 3).

**Figure 5:**
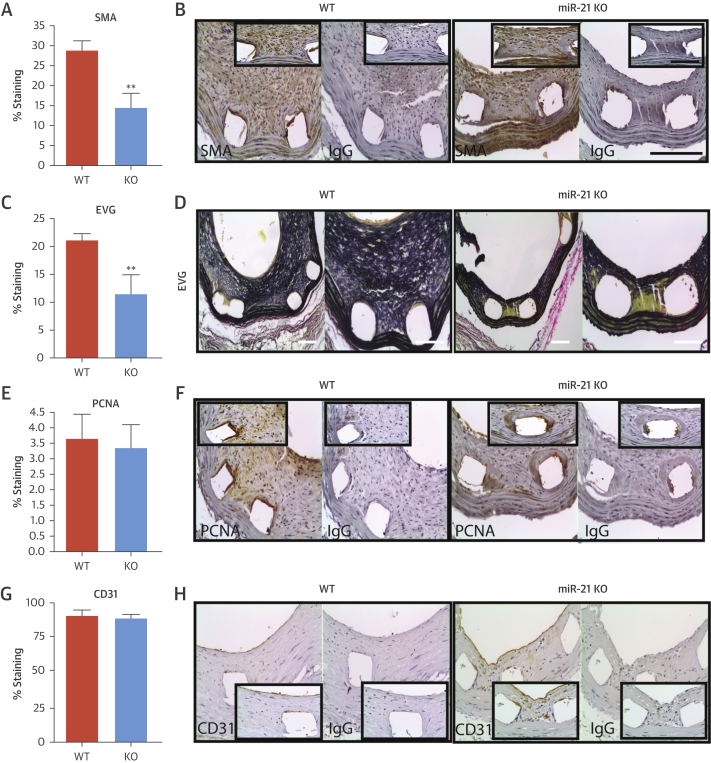
Cellular Analysis of Murine Lesions **(A and B)** The cellular composition of the neointimal lesions was quantified in WT and miR-21 KO mice at 28 days post-stenting, showing quantification of: the percentage of SMA-positive cells and representative image of the immunohistochemistry; **(C and D)** elastin Van Gieson (EVG) staining and representative images; **(E and F)** percentage of cells staining positive for proliferating cell nuclear antigen (PCNA); and **(G and H)** percentage of cell staining positive for CD31 within the circumference of the lumen and representative images. **p < 0.01 versus WT mice (Student unpaired *t* test). **Scale bar** = 100 μm. Abbreviations as in Figure 2, Figure 4.

The neointimal lesions from miR-21 KO mice contained significantly less α-actin–positive SMCs (28 ± 2.4% [n = 8] vs. 14 ± 3.7% [n = 9]; p < 0.01) (Figures 5A and 5B). Lesions in WT mice contained 50% more elastin than in KO mice (Figures 5C and 5D, Online Table 2). No difference in cell proliferation was observed after quantification of cells in the neointima (Figures 5E and 5F, Online Table 2). We assessed vessel reendothelialization and, importantly, found no significant difference between miR-21 KO and WT mice (91.0 ± 3.9% vs. 88.7 ± 2.9%, respectively) (Figures 5G and 5H). No differences were observed at baseline (i.e., unstented vessels) (Online Table 3).

**Figure 6:**
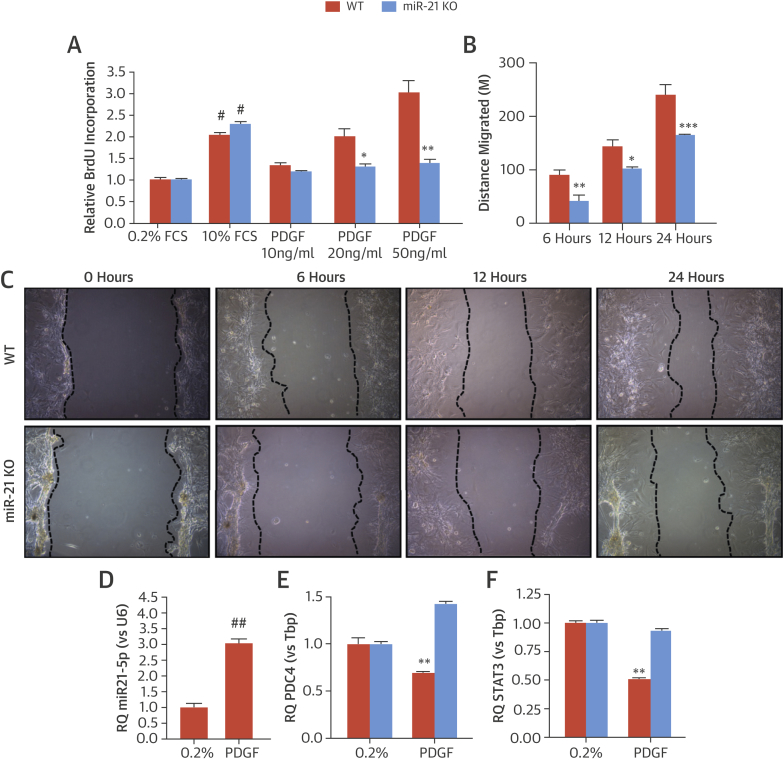
In Vitro Analysis of miR-21 SMC Proliferation and migration of vascular smooth muscle cells (SMCs) were studied via proliferation assay by using **(A)** bromodeoxyuridine (BrdU) incorporation relative to 0.2% fetal calf serum (FCS) and **(B)** distance migrated by WT and KO aortic SMCs in response to platelet-derived growth factor (PDGF) 6, 12, and 24 h after stimulation. **(C)** Photographs represent miR-21 WT and KO SMC migration. **(D)** miR-21 expression levels are seen in SMCs stimulated with PDGF relative to quiesced cells (0.2% FCS). **(E)** Relative expression of programmed cell death protein-4 (PDCD4) and **(F)** signal transducer and activator of transcription 3 (STAT3) in miR-21 WT and KO SMC is significantly stimulated with PDGF. Relative quantitation (RQ) ± rqmax (vs. TATA-binding protein [Tbp]). **(D)** #p < 0.05 and ##p < 0.01 vs 0.2% FCS (Student unpaired *t* test) or **(A, B, E, and F)** *p < 0.05; **p < 0.01; ***p < 0.001 versus WT cells (2-way analysis of variance with Bonferroni post-hoc test). Abbreviations as in Figure 4.

To explore the mechanisms responsible for the reduction in neointima formation in miR-21 KO mice, proliferation and wound healing assays were performed. The proliferative response of miR-21 KO SMC was dramatically attenuated in response to platelet-derived growth factor (PDGF) (Figure 6A), as was migration in response to PDGF (Figures 6B and 6C). To identify potential targets responsible for these effects, we stimulated aortic SMC from miR-21 WT and KO mice with PDGF (n = 5) to elevate miR-21 levels (3.03 ± 0.23-fold vs. controls; p < 0.01) (Figure 6D). The messenger RNA levels of known miR-21 targets with defined roles in SMC proliferation and migration were profiled. The expression of programmed cell death protein 4 (PDCD4) and signal transducer and activator of transcription 3 were significantly reduced in aortic SMC from WT mice after PDGF stimulation. These changes were not observed in miR-21 KO cells stimulated with PDGF (Figures 6E and 6F); however, further experiments are required to determine whether repression of these genes is direct or indirect via miR-21 regulation in this setting.

**Figure 7:**
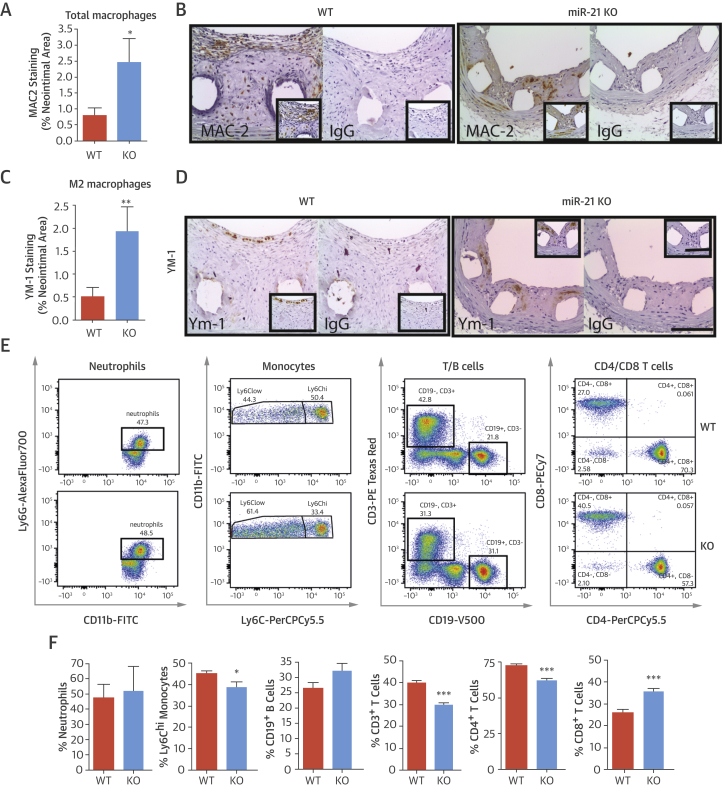
Inflammatory Cells in Neointimal Lesions and Blood of miR-21 KO Mice **(A)** Quantification and **(B)** representative images of total galactose-specific lectin 3 (MAC2) staining (% neointimal area) are seen in sections of stented graft from WT and miR-21 KO mice 28 days post-stenting (**scale bar** = 100 μM). **(C)** Quantification and **(D)** representative images of total chitinase 3–like 3 (YM-1) staining (marker for M2 macrophages) (% neointimal area) are seen in sections of stented graft from WT and miR-21 KO mice at day 28. Flow cytometric assessment of circulating cells in blood of WT and miR-21 KO mice. **(E)** Representative fluorescence-activated cell sorting plots and **(F)** bar charts showing percent quantification of cells in gate potentially indicate a reduced ability to develop proinflammatory responses. Gating markers used: neutrophils (CD45+Ly6G+CD11b+), monocytes (CD45+Ly6G-Ly6C+CD11b+), B cells (CD45+CD3-CD19+), and T cells (CD45+CD3+CD4+ or CD45+CD3+CD8+). *p < 0.05; **p < 0.01; ***p < 0.001 versus WT mice (Student unpaired *t* test). Abbreviations as in Figure 2, Figure 4.

MiR-21 KO mice contained greater numbers of galactin-3+ (MAC-2) macrophages in the neointima compared with WT mice (0.79 ± 0.23% vs. 2.54 ± 0.75%; p < 0.05) (Figures 7A and 7B) and enhanced levels of YM-1–positive macrophages, a validated murine marker of the alternatively activated (M2) macrophage (1.93 ± 0.54% vs. 0.50 ± 0.21%; p < 0.01) (Figures 7C and 7D). Thus, loss of miR-21 results in altered inflammatory cell phenotype within injured vessels. To investigate whether these effects were derived from any hematological defect, before cell recruitment to the vessel wall, the populations of immune cells in both bone marrow and blood of WT and miR-21 KO mice were examined (16). In bone marrow, the percentage of cluster of differentiation (CD) 3+ T cells was significantly reduced in miR-21 KO mice, but neutrophils, monocytes, B cells, CD4+, and CD8+ T cells did not differ (Online Figures 3A and 3B). In blood, miR-21 KO mice exhibited a significantly reduced percentage of circulating Ly6c+hi monocytes and CD3+ T cells (Figures 7E and 7F), potentially indicating a reduced capacity to develop proinflammatory responses.

**Figure 8:**
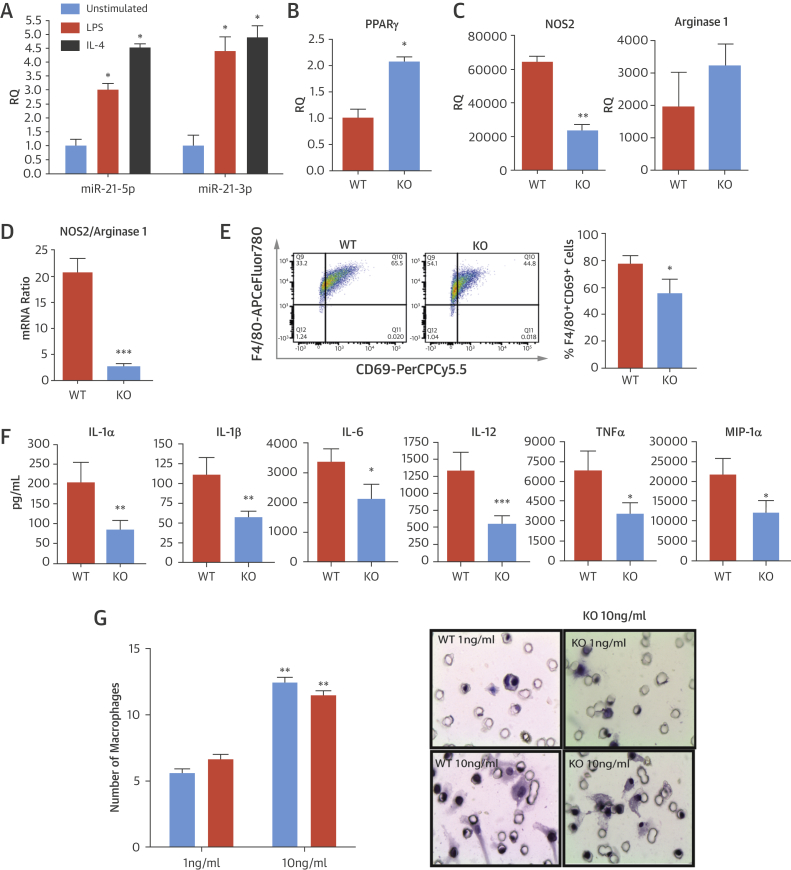
In Vitro Altered Inflammatory Response in miR-21–Deficient Macrophages **(A)** RQ of expression of miR-21-5p and miR-21-3p in WT macrophages stimulated with either lipopolysaccharide (LPS) or interleukin (IL)-4 for 20 h; data normalized to U6. Expression of peroxisome proliferator–activated receptor (PPAR)-γ messenger ribonucleic acid (mRNA) by quantitative polymerase chain reaction in **(B)** unstimulated (baseline) macrophages and **(C)** nitric oxide synthase (NOS2) and arginase 1 mRNA in LPS-stimulated macrophages from WT and miR-21 KO mice. Data are normalized to Tbp. **(D)** Ratio of expression of NOS2/arginase mRNA by quantitative polymerase chain reaction in LPS-activated macrophages was significantly higher in WT mice than in miR-21 KO mice. **(E)** Flow cytometric assessments found that the cell surface marker CD69 was higher in WT versus miR-21 KO macrophages after stimulation with LPS for 20 h. Representative fluorescence-activated cell sorting plot and bar chart showing quantification of data (% of F4/80+ cells expressing the marker). **(F)** Inflammatory cytokine (IL-1α, IL-1β, IL-6, IL-12, and TNF-α) and chemokine (macrophage inflammatory protein [MIP]-1α) production in LPS-activated macrophages was reduced in miR-21 KO compared with WT mice. **(G)** In a macrophage invasion and migration assay, the number of bone marrow–derived macrophages migrating though Matrigel-coated transwell inserts in response to monocyte chemoattractant protein (MCP)-1 was fixed at 6 h and quantified. Representative images of membranes are shown adjacent to graph. *p < 0.05; **p < 0.01; ***p < 0.001 versus WT mice (Student unpaired *t* test). Abbreviations as in Figure 2, Figure 4, Figure 6.

To investigate whether the absence of miR-21 leads to altered macrophage differentiation, BMDMs from WT and miR-21 KO mice were generated and treated with either lipopolysaccharide (LPS) or interleukin (IL)-4 in vitro to induce M1 and M2 polarization, respectively. Both LPS and IL-4 significantly up-regulated the expression of miR-21-3p and miR-21-5p in WT macrophages (3.00 ± 0.23-fold, 4.38 ± 0.91-fold, 4.74 ± 0.13-fold, and 4.88 ± 0.7-fold, respectively; p < 0.05), indicating that inflammatory mediators can modulate the expression of miR-21 (Figure 8A). At baseline, unstimulated (M0, nonpolarized) miR-21 KO macrophages had significantly higher levels of peroxisome proliferator–activated receptor (PPAR)-γ expression (M2 polarization marker) than WT cells (p < 0.01) (Figure 8B). After activation with LPS, expression of nitric oxide synthase (an M1 marker) was significantly reduced in miR-21 KO versus WT macrophages (Figure 8C). In addition, the ratio of messenger RNA expression of nitric oxide synthase, compared with arginase 1 (an M2 marker), was significantly higher in WT than miR-21 KO macrophages treated with LPS (20.6 ± 6.52-fold vs. 2.7 ± 1.24-fold; p < 0.001) (Figure 8D). Flow cytometric analysis demonstrated that after activation with LPS, CD69 was significantly reduced in miR-21 KO versus WT LPS-treated macrophages (1.8-fold; p < 0.05) (Figure 8E). However, all other markers examined by using flow cytometry (major histocompatibility complex-II, CD11c, CD86, CD206, and Toll-like receptor-2) were not significantly different in either LPS- or IL-4–treated WT or miR-21 KO BMDM (Online Figure 4). The levels of several proinflammatory mediators were significantly reduced from LPS-stimulated macrophages of miR-21 KO compared with WT mice: IL-1α, IL-1β, IL-6, IL-12, tumor necrosis factor-α, and macrophage inflammatory protein-1α (Figure 8F). In addition, the IL-12/IL-10 ratio for LPS-stimulated miR-21 macrophages was almost double that of KO macrophages (24 vs. 13).

To address whether miR-21 KO macrophages had a reduced capacity to infiltrate and migrate into vascular lesions after injury, we studied BMDM migration through Matrigel-coated transwell inserts containing 8-μm pores (Sigma, United Kingdom). These experiments showed that both BMDM isolated from WT and miR-21 KO mice had the same capacity to migrate through a matrix membrane in response to monocyte chemoattractant protein-1 (Figure 8G).

## Discussion

We report for the first time miRNA patterns associated with delayed arterial healing and neointima formation after stenting (Central Illustration). Aberrantly expressed miRNA lead and “passenger” strands associated with 7- and 28-day time points were identified. Of particular interest, the stem loop of miR-21, including lead (miR-21-5p) and passenger (miR-21-3p) strands, was up-regulated in both the BMS and DES groups compared with controls. Further experiments highlighted that loss of the miR-21 stem loop in KO mice blocked neointimal formation through effects on SMC proliferation and migration, macrophage polarization, and inflammatory activation. A causal network analysis was performed of miR-21-3p and miR-21-5p targets to determine whether these miRNAs affect the inflammatory networks associated with vascular injury. Despite a relatively small overlap of predicted genes, pathways associated with tumor necrosis factor-α and IL-1 were 1 of the top-ranking regulatory networks (Online Figure 5); this finding may be relevant because both of these cytokines play prominent roles in neointima formation 17, 18.

**Central Illustration:**
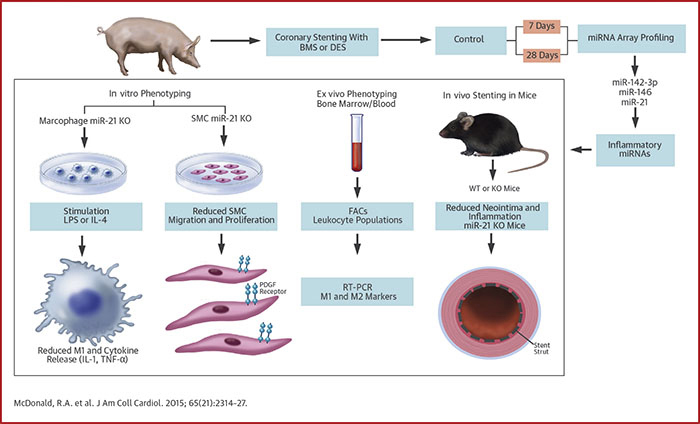
Role of miRNA in ISR Although drug-eluting stents (DES) reduce the incidence of in-stent restenosis (ISR), they delay vascular healing and are associated with a chronic inflammatory response, which involves micro–ribonucleic acids (miRNAs). In pig, mouse, and in vitro models, miR-21 promotes vascular inflammation and remodeling *after* stenting and may be a therapeutic target to enhance wound healing after vascular injury. BMS = bare-metal stent(s); FACS = fluorescence-activated cell sorting; IL = interleukin; KO = knockout; LPS = lipopolysaccharide; PDGF = platelet-derived growth factor; RT-PCR = real-time polymerase chain reaction; SMC = smooth muscle cell; TNF = tumor necrosis factor; WT = wild type.

MiR-21 KO mice exhibited reduced SMC deposition, neointima formation, and an altered inflammatory phenotype, resulting in enhanced levels of anti-inflammatory M2 macrophages in response to vascular injury and stenting, with no effect on endothelial regeneration. Subsequent profiling of immune cell populations in the blood of miR-21 KO mice demonstrated reduced numbers of Ly6c+hi cells, a cell type that can differentiate into M1 macrophages after tissue infiltration. This finding suggests that miR-21 KO mice have a reduced capacity to develop an M1 inflammatory phenotype in response to injury. We also found reduced total CD3+ T-cell counts in the bone marrow and blood of the miR-21 KO. Recently, it has been reported that miR-21 can modulate T-cell responses, including alterations in cytokine production and apoptosis rates 19, 20, 21. T cells are already known to contribute to the inflammatory response after coronary artery stenting (22); thus, the absence of miR-21 in T cells likely contributes to the altered inflammatory responses and reduced in-stent stenosis seen in miR-21 KO.

In support of the altered inflammatory responses in vivo, our BMDM experiments revealed that miR-21 KO macrophages contain enhanced baseline levels of PPAR-γ, a well-characterized M2 macrophage marker, and a reduced ratio of nitric oxide synthase/arginase 1 (M1/M2 markers). These findings are particularly relevant in the setting of inflammatory vascular disease because numerous studies suggest that PPAR-γ activation can curtail the inflammatory response. Activation of PPAR-γ in human macrophages reduces matrix metallopeptidase 9 activity and inhibits expression of IL-1β, IL-6, and tumor necrosis factor-α 23, 24, 25. Furthermore, several groups have reported that PPAR-γ agonists inhibit atherosclerosis development and reduce inflammatory markers in apolipoprotein E KO mice 26, 27. Furthermore, miR-21 KO macrophages exhibited a reduced capacity to secrete proinflammatory mediators such as IL-1, tumor necrosis factor-α, macrophage inflammatory protein-1, IL-6, and IL-12 in response to LPS, a substance known to stimulate the inflammatory M1 macrophage phenotype. However, a defective response to LPS does not necessarily mean altered polarization and may simply reflect, for example, defective CD14 expression. Further phenotyping of surface receptors from BMDM also showed reduced expression of CD69 in miR-21 KO mice. A CD69 deficiency may contribute to reduced inflammatory cytokine secretion, as previous studies have reported that CD69 activation mediates numerous inflammatory processes such as nitric oxide production and release of tumor necrosis factor-α from murine macrophages and T cells 28, 29. Thus, loss of miR-21 may accelerate wound healing and resolution of the inflammatory response after vascular injury and stenting, events that could reduce the incidence of late stent thrombosis.

It is important to note that we used human arrays in the porcine samples to identify miRNAs that would extrapolate to the pathology in the clinical setting. It is possible that a proportion of miRNAs may be underrepresented due to the sequence variation or chromosomal locations between pig and human. We identified an almost 20-fold up-regulation of miR-21-3p after stenting with BMS and DES. Our subsequent experiments in BMDM demonstrated that both miR-21-3p and miR-21-5p were up-regulated in response to LPS, which induces classical macrophage polarization (M1). In combination with reduced inflammatory cytokine release from miR-21 KO macrophages, these results suggest that miR-21-3p and miR-21-5p may both play a pathological role in macrophage activation in response to inflammatory stimuli.

Currently available DES directly target SMC proliferation to prevent neointima formation. We observed that miR-21 plays a prominent role in SMC proliferation and migration in response to vascular injury, consistent with other studies 30, 31. Aortic SMC isolated from miR-21 KO mice had a reduced capacity to migrate and proliferate in response to PDGF, an important observation because novel DES must retain the antiproliferative effect on SMC accumulation to maintain their clinical efficacy. These findings are consistent with previous reports demonstrating that pharmacological or genetic knockdown of miR-21 reduces SMC proliferation and neointima formation after balloon injury or vein grafting 30, 31, 32, 33. Several of these reports suggest that miR-21 mediates a beneficial effect on SMC proliferation and neointima, at least in part, via inhibition of phosphatase and tensin homolog.

We also found that the levels of PDCD4 were suppressed after PDGF exposure in WT mice. Importantly, the levels of PDCD4 were increased in miR-21 KO mice, suggesting that PDCD4 is modulated after exposure to PDGF and plays a role in SMC proliferation and migration, effects repressed in miR-21 KO SMCs. In concordance with these findings, previous studies suggest that PDCD4 is down-regulated after vascular injury, and overexpression of PDCD4 with adenoviral vectors increases apoptosis and reduces SMC proliferation (34).

### Study limitations

Although the present study demonstrates that miR-21 play a prominent role in the pathology of in-stent restenosis, it is important to note that these studies are based on data from pre-clinical animal models of restenosis. Although our results in miR-21 knockout mice demonstrate that loss of miR-21 reduces in-stent restenosis and inflammatory cell function, it is important to note that these defects are present before vascular injury, these deficiencies may alter the response to injury in these mice. In order to demonstrate that these finding have efficacy in the clinic, further studies are required to demonstrate that pharmacological knockdown of miR-21 or miR-21 targets can inhibit neointimal formation and vessel inflammation from current drug-eluting stent platforms. Furthermore, detailed pharmacokinetic profiling would be needed to demonstrate an effect elution profile of antimiR-21 therapy, without any off-target effect.

## Conclusions

The miR-21 stem loop plays an important role in SMC and macrophage activation after vascular injury. Our findings in the murine model of ISR revealed that loss of miR-21 attenuates neointima formation and macrophage activation, resulting in a less inflammatory phenotype. These findings suggest that miR-21 modulation could enhance wound healing and resolve the inflammatory response, effects that could improve the clinical efficacy of currently available DES. The miR-21 axis warrants further investigation as a therapeutic target in the setting of stent-induced inflammatory vascular disease. Lineage-restricted knockouts maybe required to unravel the complex role of miR-21 in this setting.

Perspectives **COMPETENCY IN PATIENT CARE:** DES reduce in-stent restenosis after percutaneous coronary intervention but are associated with a greater risk of stent thrombosis due to delayed arterial healing that is characterized histologically by incomplete reendothelialization and persistent fibrin and inflammatory cell deposition. An array of microRNA molecules are involved in the inflammatory processes driving the cellular response to vascular injury, and genetic KO of the miR-21 stem loop attenuates neointimal formation after arterial stenting in mice.

**TRANSLATIONAL OUTLOOK:** Future investigations should seek to determine whether local antimiR to knockdown miR-21 levels delivered directly from drug-eluting stents could reduce vessel inflammation and neointima formation and reduce the risk of both restenosis and stent thrombosis.
