# Supplementary material for: Reducing In-Stent Restenosis: Therapeutic Manipulation of miRNA in Vascular Remodeling and Inflammation
Source: J Am Coll Cardiol. 2015 Jun 2;65(21):2314–27. doi: 10.1016/j.jacc.2015.03.549 (PMC4444526; doi:10.1016/j.jacc.2015.03.549)

## Supplementary methods

### Animals and tissues

All animal procedures were performed in accordance with UK Home Office Guidance on the operation of the Animals (Scientific Procedures) Act 1986 and institutional ethical approval (PPL60/4114 and PPL 60/4429). Male white Landrace pigs (SAC Commercial Ltd, Edinburgh, UK) and miR-21 knockout mice (1) were maintained on a 12 hour light/dark cycle with free access to food and water at a designated Biological Procedures Unit prior to stenting and maintained for a period of 7 or 28 days thereafter. Stented Human coronary arteries were dissected from explanted hearts at the time of cardiac transplantation. All procedures had local ethical approval (Research Ethical Committee number: 06/S0703/110) and experimental procedures conformed to the principles outlined in the Declaration of Helsinki.

### Porcine Coronary Stenting

Stenting of porcine coronary arteries was performed as previously described (2). Briefly, Male Landrace pigs (19-26 kg) were pre-dosed orally with aspirin and clopidogrel 24 hours prior to surgery and maintained on this dual antiplatelet therapy for the remainder of the study to reduce the risk of in-stent thrombosis. Pigs were sedated by intramuscular (IM) injection of Tiletamine/Zolazepam (Zoletil; Virbac, France). Induction of general anesthesia was conducted using intravenous (IV) Propofol (Rapinovet; Schering-Plough, Welwyn Garden City, UK) and maintained with isoflurane (Abbott Laboratories Ltd, UK). Pigs received 100IU/kg intravenous heparin (Leo Laboratories, UK) prior to commencement of the procedure. Blood pressure, oxygen saturation and ECG monitoring were conducted throughout the procedure. Vascular access was obtained by femoral artery cut down and the insertion under direct observation of a 6 Fr transradial sheath (Arrow International, UK). Coronary angiography was performed prior to the deployment of either bare metal stents (Gazelle™, Biosensors, Switzerland) or biolimus A9 eluting stents (Biomatrix Flex™, Biosensors, Switaerland) to achieve a target ratio of stent to artery diameter of 1.2:1. Pigs received buprenorphine 0.15 mg IM (Vetergesic: Alstore Ltd, UK) and ampicillin 350 mg IM (Amfipen LA, UK) immediately following the procedure.

Animals were euthanized after 7 or 28 days by intravenous overdose of euthatal and coronary vessels carefully removed. Vessels were halved with total RNA extracted from one half of the stented arteries and the remaining half fixed in 4% PFA for histological analysis. Optical Coherence Tomography (OCT) was conducted *ex vivo* on coronary arteries following vessel fixation, using Light lab and a Dragonfly™ OCT catheters (St Jude Medical, Stratford-upon-Avon, UK), the vessels were submerged under phosphate buffered saline to prevent refraction of the images *ex vivo*.

### Murine Arterial Stenting Model

Each procedure was carried out under sterile conditions as outlined previously (3). Briefly, a stainless-steel stent (5 cell, 2.5 x 0.8 mm; strut thickness 0.06 mm; Cambus Medical, Ireland) was crimped onto a 1.20 mm x 8 mm minitrek balloon angioplasty catheter (Abbott Vascular, Paisley) and deployed (10 atm for 30 seconds) into the thoracic aorta. Recipient mice were anaesthetized using a

combination of subcutaneous Hypnorm (25 mg/kg, Bayer) and Hypnovel (25 mg/kg, Roche). The right common carotid artery was mobilized free from the thoracic inlet to its bifurcation, ligated, and divided between ties at its midpoint. Polyethylene cuffs (0.65 mm diameter, Portex LtD) were placed at both ends of the artery, were everted over the cuff and secured with suture and the stented aorta was interposition grafted between the two ends of the carotid artery by sleeving the ends over the cuffed artery cuff and secured with 8/0 suture. Mice were allowed to recover in heated chambers for 24 hours and returned to normal housing conditions and maintained on aspirin supplemented water and normal chow diet for a further 28 days. To reduce the risk of in-stent thrombosis (IST), one week prior to surgery each mouse was pre-dosed with aspirin supplemented drinking water at a concentration of 300mg/L and continued until the end of the experiment. At harvesting animals were euthanized and transcardial perfusion with heparinized saline performed. The stented graft was removed and fixed for 12 hours in 4% Paraformaldehyde (PFA).

**Electrolysis**

Following fixation in paraformaldehyde the stented murine arteries were immersed in 5% (w/v) citric saline and electrochemical dissolution performed by passing a small direct current through the stent as described previously (4).

**Immunohistochemistry**

For histological analysis, vessels were fixed in 4% paraformaldehyde for 24 hours. Murine stented aortas were electrolyzed to remove the stents, as described above, dehydrated in graded ethanol cleared in Histo-Clear and embedded in paraffin wax, before sectioning and subsequent staining. In brief serial paraffin sections were dewaxed and rehydrated. Tissue sections were subjected to citric acid antigen retrieval and endogenous peroxidase activity was inhibited by incubation with 3% hydrogen peroxide. After blocking sections with 20% (vol/vol) rabbit or goat serum in PBS, sections were incubated overnight at $4^0$C with purified rat mAb against mouse macrophages (mac-2; BD Biosciences, Oxford, UK) at 3.12μg/ml, mouse mAb against α-smooth muscle actin (Sigma) at 82μg/ml in 1% (wt/vol) BSA in PBS, proliferating cell nuclear antigen at 2μg/ml (PCNA, Abcam, Cambridge, UK), rat anti-mouse CD-31 (Dianova, Germany) at 5μg/ml or Rabbit anti-mouse YM-1 (Stem Cell technology) at 3.2μg/ml or mouse anti human CD68 (DAKO, UK) at 2μg/ml. Sections were then incubated with the appropriate biotinylated secondary antibody (DAKO) diluted 1:200 in 3% (vol/vol) goat serum in PBS, and then horseradish peroxidise labelled Extravidin (diluted 1:400 in 1% (wt/vol) BSA in PBS). Optimal visualization of staining was achieved using 3',3'-diaminobenzidine tetrahydrochloride dihydrate and hydrogen peroxide. Sections were counter-stained with hematoxylin. A negative control, where the primary antibody was replaced with mouse, rat or rabbit IgG at the same dilution, was routinely included. All immunohistochemistry staining was quantified as the area of positive staining expressed as a percentage of total neointimal area.

***In situ* hybridization**

*In situ* hybridization was carried out on routinely processed tissue sections which were rehydrated as described above. These tissue sections were then treated with 0.5U/ml proteinase K (Sigma) at 37 degrees for 15 minutes, then fixed with 4% paraformaldehyde for a further 10 minutes. After washing with phosphate buffered saline (PBS), slides were incubated with hybridization buffer for 1h at

52 or 58 degrees for miR-21 and scramble probe, respectively. Slides were then hybridized with 40nM double DIG-labelled miR-21 or double DIG-labelled scramble probe (Exiqon) at 52 or 58 degree overnight. After washing and blocking, slides were incubated with anti-DIG-AP Fab fragments (Roche) in blocking buffer at 4 degrees overnight, and washed with PBST (PBS plus 0.1% Tween 20, Sigma) and AP buffer (0.1M Tris-HCL pH9.5, 150mM NaCL, 5mM MgCL2, 0.1% Tween20). MiR-21 was visualized with BM purple solution (Roche) for 16-24 hrs at room temperature until the staining was visible under microscope.

**Vessel storage and RNA isolation**

Harvested porcine coronary arteries were placed in RNA*later*®-ice (Invitrogen, Paisley, UK Paisley, UK) and stored at -80$^0$C until the day of isolation. RNA from arteries was isolated following disruption of the vessels under liquid nitrogen using a pestle and mortar, and these vessel fragments were placed in pre-cooled Qiazol (Invitrogen, Paisley, UK) and homogenized using a tissue homogenizer (Polytron, Switzerland). The RNA was processed through miRNeasy kit (Qiagen, Hilden, Germany) following the manufacturer's instructions, treated with DNAse 1 (amplification grade; Sigma, St. Louis, MO, USA) in order to eliminate genomic DNA contamination. The yield and purity of RNA were measured using a NanoDrop ND-1000 Spectrophotometer (Nano-Drop Technologies, Wilmington, DE, USA), and RNA integrity was assessed using the RNA 6000 Nano LabChip kit (Agilent Technologies).

**Global MicroRNA Expression Profiling**

The global profiling for miRNAs in control un-stented arteries and stented porcine coronary arteries was performed using the TaqMan Low-Density Array Human MicroRNA Panel v2.3 (Applied Biosystems, CA, USA), which included Cards A and B in a 384 well format. The A cards contained 377 human miRNAs and 3 endogenous controls, B cards contained 287 miRNA and 6 endogenous control miRNAs, these cards were experimentally processed following manufacturer's instructions. In brief, total RNA (100 ng) was first reverse-transcribed with the Multiplex RT pool set A or B (Applied Biosystems, CA, USA) through a reverse transcription (RT) step using the High-Capacity cDNA Archive kit (Applied Biosystems, CA, USA), wherein a stem-loop RT primer specifically binds to its corresponding miRNA and initiates its reverse-transcription. The RT mix included 50 nM stem-loop RT primers, 1 x RT buffer, 0.25 mM each dNTPs, 10 U/µl MultiScribe reverse transcriptase, and 0.25 U/µl RNase inhibitor. The 7.5 µl reaction mixture was then incubated for 30 min at 16$^0$C, 30 min at 42$^0$C, 5 min at 85$^0$C, and then held at 4$^0$C. The RT products were subsequently amplified with sequence-specific primers; using the Applied Biosystems 7900 HT Real-Time PCR system. Six µl of RT product were added to 444 µl nuclease-free water and mixed with 450 µl Taqman Universal Master Mix II, No UNG, then dispensed into a 384 well plate by centrifugation. The reactions were incubated in the plate at 95$^0$C for 10 min followed by 40 cycles of 95$^0$C for 15 sec and 60$^0$C for 1 min.

For analysis, fold-changes for each miRNA were normalized to U6 since this miRNA was the most suitable endogenous miRNA in porcine tissue. The relative expression levels between samples were calculated using the comparative delta *C*t (threshold cycle number) method with a control sample (normal) as the reference point. Data analysis was performed by using the SDS software version 2.3 (Applied Biosystems, CA, USA) and the baseline and threshold were automatically set. Data were normalized and then analyzed to identify miRNAs that are differentially expressed between the control (un-stented arteries) and arteries subjected to

stenting for a period of 7 or 28 days. Data were analyzed using Data Assist analysis software, version 3 (Applied Biosystems, CA, USA).

**miRNA quantification by real-time polymerase chain reaction (qRT-PCR)**

cDNA was synthesized from RNA using stem-loop reverse transcription primers (applied biosystems, Foster City, CA, USA). qRT-PCR was performed using TaqMan® universal master mixII with Taqman microRNA expression probes according to the manufacturer's instructions. Expression was normalized to U6 or Tbp for microRNA or target expression data, respectively and expressed as relative expression levels of miRNAs or mRNAs of interest as described in (5). Results are shown relative to the experimental control using the -2ΔΔCt method described by Livak (6). Assay information available on request.

**Proliferation and migration assays**

Murine aortas were harvested from male mice aged 8 - 12 weeks Vascular SMCs were isolated and cultured as described by Johnstone *et al* (7). Briefly aortic SMC were plated at sub-confluence in serum depleted medium before cells were subjected to scratch wounds with a 200ul pipette tip and incubation with 15% fetal calf serum of PDGF-BB for a period of 24hrs. Proliferation of VSMCs was assessed using a commercially available 5-Bromo-2'-deoxyuridine (BrdU) assay as per manufacturer's protocol (Millipore, Livingstone, UK). Murine VSMCs, from miR-21 WT and KO mice, passage 4 to 6 were seeded into 96 well plates at a density of $7x10^3$ cells/well in DMEM with 10% FCS and incubated overnight at 37ºC in the presence of 5% $CO_2$. VSMCs were quiesced for 24 hours in DMEM with 0.2% FCS. Following quiescence cell proliferation was stimulated by the replacement of media containing one of four experimental conditions: 10% FCS, Platelet Derived Growth Factor-ββ (PDGF- ββ) (R&D Systems, Abingdon, UK) 10ng/mL, PDGF- ββ 20 ng/mL or PDGF- ββ 50 ng/mL. 0.2% FCS was used as control. All experimental conditions were conducted in biological triplicate and technical duplicate. Experiments were terminated at 48 hours.

Scratch assay was utilized to assess cell migration as described previously (8). Briefly, VSMCs isolated from miR-21 WT and KO mice were seeded into 12 well plates at a density of $1x10^5$ cells per well and grown in DMEM with 10% FCS, at 37ºC in the presence of 5% $CO_2$ until fully confluent. The cells were quiesced as described previously. Horizontal lines were drawn on the under surface of each well to act as a reference for measurements. Three straight, scratches perpendicular to the reference lines were incised in the cell monolayer of each well using a sterile 200 µl pipette tip. Cellular debris was removed by gently washing cells once with 1 mL of sterile PBS. Cells were incubated with DMEM containing 0.2% FCS, 10% FCS or PDGF- ββ 20 ng/mL.

**Generation of Bone Marrow-Derived Macrophages**

Bone Marrow Derived Macrophages (BMDM) were generated as described previously (9). Briefly, bone marrow cells were isolated from femurs and tibiae of WT and miR-21 KO mice. Cells were filtered through a 100 µm filter and then spun at 300 *g* for 5 minutes. Following treatment with Red Cell Lysis Buffer (Sigma) cells were grown for 6 days in non-bacteriological petri dishes ($2x10^6$ per

plate) in 10mls complete media (RPMI-1640/10% heat-inactivated fetal bovine serum/2mM L-glutamine, 100 units/ml penicillin and 100 µg/ml streptomycin) containing 50 ng/mL recombinant murine macrophage colony stimulating factor (M-CSF, Peprotech). Media was refreshed on day 3 and on day 6 cells were scraped off using a cell scraper, counted and plated out at $0.5x10^6$ cells/well on a 24 well plate prior to activation with IL-4 (2 ng/ml, Peprotech) or Lipopolysaccharide (LPS) (100 ng/ml, Bacterial LPS from E. coli, Serotype O111:B4 TLRgrade™, EnzoLifeSciences) for 20 hours.

## In vitro invasion studies on BMDM from WT and miR-21 KO mice

Migration of BMDM was measured by the invasion of cells through Matrigel-coated Corning™ transwell inserts (Sigma). Transwell inserts containing 8µm pores were coated with Matrigel (40µL/well; BD Biosciences, UK). Cell suspensions (200µL; $1x10^5$ cells) in RPMI 1640medium (supplemented with 100IU/ml penicillin, 100µg/mL streptomycin and 2mmol/L L-glutamine) was added to the upper chamber. The same medium (600µL) supplemented wit 1 or 10ng/mL of murine recombinant monocyte chemoattractant protein-1 (MCP-1) (R&D Systems, Abington, UK) was placed in the lower wells. Cultures were incubated for 6 hours and the cells on the under surface of the transwell membrane were fixed with 4% paraformaldehyde, stained with haematoxylin, counted in 10 random fields.

## Flow cytometry

Bone marrow cells were isolated from femurs and tibiae of WT and miR-21 KO mice and blood collected by cardiac puncture. Cells were treated with 1 ml of Red Blood Cell Lysis Buffer (StemCell Technologies) for 20 min on ice. Cells were then treated with Fc receptor block (CD16/CD32) (BD Pharmingen) at 4 degrees for 20 mins. To identify myeloid cells in blood and bone marrow, cells were stained for 30 mins at 4°C with fluorescent antibodies against: CD45-PECF594, Ly6C-PerCPCy5.5, CD206-PE (all BD Biosciences); Ly6G-AF700, MHC II-PECy7 (all eBioscience). To identify T cells in bone marrow and blood, cells were stained with: CD45-FITC, CD4-PerCPCy5.5, CD8-PECy7 (all eBioscience); CD3-PECF594, CD19-V500 (both BD Biosciences). For macrophage *in vitro* studies cells were collected 20 hrs after polarisation and treated with Fc receptor block, then stained with fluorescent antibodies against: F4/80-APCefluor780, MHC II-PECy7, TLR2-PE, and CD86-FITC (all e-bioscience); CD206-APC (BioLegend); CD11c-FITC and CD69-PerCPCy5.5 (both BD Biosciences) for 30 minutes. Cells were washed once and then flow cytometry performed using a BD FACS Canto™ II or a LSR II (BD Biosciences). All flow analysis was conducted using FlowJo v10 (Treestar).

## Luminex Assay

Macrophage culture supernatants were collected and analyzed on a Cytokine Mouse 20-Plex Panel (Life Technologies) according to the manufacturer's instructions.

## Statistics

All data are mean ± standard error of mean (SEM). For the comparison of mean values a Bartlett's test for equal variances was performed - there was no evidence of heterogeneous variances between groups for any of the comparisons. Visual assessment was used to check for any lack of normality; as there was no evidence of this, one way ANOVA followed by a Tukey's multiple comparison test (for comparison of more than two groups) or Students t-test (for comparison of two groups) were carried out. For all the q-PCR experiments, values are expressed as fold change. All statistical analysis using Graph Pad Prism v4 (GraphPad Software®). The microRNA array data were analyzed in DataAssist™ software (Life Technologies). Comparisons of *in vitro* SMC proliferation and migration were performed by 2-way ANOVA and Bonferonni's post-hoc test.


1. Patrick DM, Montgomery RL, Qi X et al. Stress-dependent cardiac remodeling occurs in the absence of microRNA-21 in mice. J Clin Invest 2010;120:3912-6.
2. Ialenti A, Grassia G, Gordon P et al. Inhibition of in-stent stenosis by oral administration of bindarit in porcine coronary arteries. Arterioscl Throm Vasc 2011;31:2448-54.
3. Ali ZA, Alp NJ, Lupton H et al. Increased in-stent stenosis in ApoE knockout mice: insights from a novel mouse model of balloon angioplasty and stenting. Arterioscl Throm Vasc 2007;27:833-40.
4. Bradshaw SH, Kennedy L, Dexter DF, Veinot JP. A practical method to rapidly dissolve metallic stents. Cardiovasc Pathol 2009;18:127-33.
5. McDonald RA, White KM, Wu J et al. miRNA-21 is dysregulated in response to vein grafting in multiple models and genetic ablation in mice attenuates neointima formation. Eur Heart J 2013;34:1636-43.
6. Livak KJ, Schmittgen TD. Analysis of relative gene expression data using real-time quantitative PCR and the 2(-Delta Delta C(T)) Method. Methods 2001;25:402-8.
7. Johnstone SR, Kroncke BM, Straub AC et al. MAPK phosphorylation of connexin 43 promotes binding of cyclin E and smooth muscle cell proliferation. Circulation research 2012;111:201-11.
8. Liang CC, Park AY, Guan JL. In vitro scratch assay: a convenient and inexpensive method for analysis of cell migration in vitro. Nat Protoc 2007;2:329-33.
9. Banerjee S, Xie N, Cui H et al. MicroRNA let-7c regulates macrophage polarization. J Immunol (Baltimore, Md : 1950) 2013;190:6542-9.

**Supplementary Table 1- miRNA regulation post stenting in pig coronary arteries**

| miRNA Up-regulated | BMS (7 Days) | | DES (7 Days) | | BMS (28 Days) | | DES (28 Days) | |
|---|---|---|---|---|---|---|---|---|
| | **Fold Change** | **P-Value** | **Fold Change** | **P-Value** | **Fold Change** | **P-Value** | **Fold Change** | **P-Value** |
| miR-21 | 2.2 | 0.172 | 4.1 | 0.008 | 4.8 | 0.054 | 3.4 | 0.012 |
| miR-21-3p | 18.9 | 0.003 | 22.2 | 0.001 | 2.7 | 0.130 | 3.8 | 0.116 |
| miR-27a-3p | 1.5 | 0.069 | 2.8 | 0.001 | 1.1 | 0.007 | 1.1 | 0.591 |
| miR-142-3p | 3.9 | 0.029 | 7 | 0.014 | 3.4 | 0.164 | 1.5 | 0.461 |
| miR-142-5p | 2.6 | 0.030 | 4.7 | 0.025 | 2 | 0.310 | 1.1 | 0.815 |
| miR-146a | 0.7 | 0.150 | 0.7 | 0.228 | 1.5 | 0.460 | 0.8 | 0.450 |
| miR-146b-5p | 1.3 | 0.180 | 5.4 | 0.005 | 2.7 | 0.034 | 3.8 | 0.001 |
| miR-155 | 1.7 | 0.220 | 4 | 0.014 | 1.9 | 0.090 | 1.9 | 0.048 |
| miR-221-5p | 2.1 | 0.043 | 5.7 | 0.002 | 2.6 | 0.344 | 1.5 | 0.353 |
| miR-221-3p | 0.53 | 0.567 | 9.48 | 0.017 | 8.57 | 0.056 | 4.9 | 0.003 |
| miR-222-5p | 1.49 | 0.103 | 2.8 | 0.004 | 1.1 | 0.606 | 1.2 | 0.218 |
| miR-222-3p | 4.95 | 0.020 | 19.1 | 0.0002 | 5.27 | 0.013 | 3.85 | 0.006 |
| miR-223-3p | 1.2 | 0.600 | 5.5 | 0.0001 | 1.4 | 0.045 | 2.8 | 0.0001 |
| **miRNA** | **Fold Change** | **P-Value** | **Fold Change** | **P-Value** | **Fold Change** | **P-Value** | **Fold Change** | **P-Value** |

| **Down-regulated** | | | | | | | | |
|---|---|---|---|---|---|---|---|---|
| let-7e | 0.6 | 0.008 | 0.7 | 0.480 | 1.6 | 0.150 | 0.5 | 0.339 |
| miR-9-3p | 0.31 | 0.002 | 0.98 | 0.949 | 0.74 | 0.018 | 0.57 | 0.038 |
| miR-20a-3p | 0.4 | 0.027 | 1.2 | 0.596 | 0.7 | 0.090 | 0.9 | 0.589 |
| miR-24-1-3p | 0.32 | 0.044 | 0.6 | 0.084 | 0.6 | 0.130 | 0.4 | 0.011 |
| miR-30e-3p | 0.3 | 0.001 | 0.5 | 0.064 | 0.5 | 0.009 | 0.6 | 0.011 |
| miR-99b | 0.4 | 0.010 | 0.6 | 0.169 | 0.9 | 0.970 | 0.5 | 0.082 |
| miR-125a-5p | 0.4 | 0.002 | 0.5 | 0.007 | 0.8 | 0.680 | 1.1 | 0.644 |
| miR-125b-1-3p | 0.5 | 0.017 | 0.8 | 0.449 | 0.8 | 0.170 | 0.9 | 0.696 |
| miR-143-3p | 0.18 | 0.037 | 0.8 | 0.887 | 1.29 | 0.590 | 0.54 | 0.184 |
| miR-145-3p | 0.27 | 0.005 | 0.67 | 0.339 | 0.4 | 0.004 | 0.42 | 0.001 |
| miR-181a | 0.4 | 0.020 | 0.8 | 0.642 | 1.64 | 0.600 | 0.6 | 0.251 |
| miR-214-3p | 0.35 | 0.023 | 0.71 | 0.697 | 0.77 | 0.400 | 0.82 | 0.622 |
| miR-424-3p | 0.2 | 0.002 | 0.6 | 0.181 | 0.5 | 0.017 | 0.6 | 0.047 |

Tabulation of the fold change (up or down-regulation) in inflammatory miRNA expression 7 and 28 days post implantation, relative to control un-stented arteries. The table also summaries the p-value of each miRNA in the D=BMS and DES groups at 7 and 28 days.

**Supplementary Table 2. Morphometric analysis of stented murine vessels.**

| | **WT mice** | **miR-21 KO mice** | **p-Value** |
|---|---|---|---|
| Total vessel Area, $mm^2$ | 0.52±0.03 | 0.55±0.3 | N/A |
| Neointimal (N) Thickness | 0.092±0.009 | 0.057±0.012 | p<0.01 |
| Neointimal Area, $mm^2$ | 0.18±0.02 | 0.12±0.02 | p<0.05 |
| Medial (M) Area | 0.09+0.005 | 0.08+0.005 | N/A |
| N/M ratio | 2.11±0.17 | 1.37±0.18 | p<0.05 |
| Lumen Area, $mm^2$ | 0.22±0.02 | 0.3±0.02 | p<0.01 |
| % Stenosis | 41.7±2.9 | 24.1±2.6 | p<0.01 |
| Stent strut depth | 0.072±0.011 | 0.03±0.007 | p<0.05 |
| Schwartz Injury Score | 0.33±0.14 | 0.44±0.15 | N/A |
| SMC, % Neointima | 28±2.4 | 14±3.7 | p<0.01 |
| Elastin Staining | 21.1±1.2 | 11.5±3.4 | p<0.01 |
| PCNA, % Neointima | 3.6±0.8 | 3.3±0.7 | N/A |
| % Re-endothelialization | 91±3.9 | 88.7±2.9 | N/A |

**Supplementary Table 3. Baseline morphometric analysis of miR-21 KO and WT vessels**

| **Day 0** | **WT (n=9)** | **KO (n=3)** | **p value** |
|---|---|---|---|
| | **Mean (SEM)** | **Mean (SEM)** | |
| Total Vessel Area ($mm^2$) | 0.58 (0.09) | 0.45 (0.05) | ns |
| Luminal Area ($mm^2$) | 0.46 (0.07) | 0.39 (0.05) | ns |
| Medial Area ($mm^2$) | 0.11 (0.03) | 0.06 (0.04) | ns |
| Medial Thickness ($mm^2$) | 0.035) 0.005) | 0.025 (0.001) | ns |
| Vessel Injury (Schwartz Score) | 0.58 (0.15) | 0.52 (0.06) | ns |

**Supplemental Figure 1.** The Venn diagrams identified the common miRNAs which are differentially expressed following direct comparison of control (un-stented arteries) to arteries stented with a bare metal stent (BMS) or drug eluting stent (DES) for a period of 7 or 28 days. The summary below highlights miRNAs implicated in SMC or vascular inflammation.

**Supplemental Figure 2. In situ hybridization for miR-21 in stented human coronary arteries.** *In-situ* hybridization for miR-21 and scrambled control in stented human coronary arteries and immunohistochemistry for SMC actin and CD68 for smooth muscle cells and macrophages, respectively. Panels; F-J are higher magnification images of the hatch regions in panels A-E. Scale bar represents 100μm.

**Supplemental Figure 3.** Flow cytometric assessment of cells in bone marrow (BM) of WT and miR-21 KO mice (n=4). Representative FACS plots (A) and bar charts showing % quantification of cells in gate (B). Gating markers used: Neutrophils ($CD45^+Ly6G^+MHC\text{-}II^-$), Monocytes ($CD45^+Ly6G^-Ly6C^+CD11b^+MHC\text{-}II^-$), B cells ($CD45^+CD3^-CD19^+$), T cells ($CD45^+CD3^+CD4^+$ or $CD45^+CD3^+CD8^+$). * $p<0.05$ vs WT mice, Students unpaired t-test.

**Supplemental Figure 4.** Flow cytometric assessment of the cell surface marker MHC-II, CD11C, CD86, CD206 and TLR-2 in WT and miR-21 KO BMDM following stimulation with LPS (100 ng/ml) (**A & B**) or IL-4 (2 ng/ml) (**C & D**) for 20 hours. Representative FACS plots and bar charts showing quantification of data (% of $F4/80^+$ cells expressing the marker, (n=9).

**[illegible]**
[illegible] Upstream regulatory and causal network analysis on predicted gene targets using Ingenuity Pathway Analysis. TNF-α was identified as a master regulator for predicted gene targets of (A) mir-21-5p and (B) mir21-3p. Known connections of both miRNAs to target genes are indicated in light blue. The letters on the relationship lines represent functional affects with the number of references to support the connections.

Supplementary Figure 1

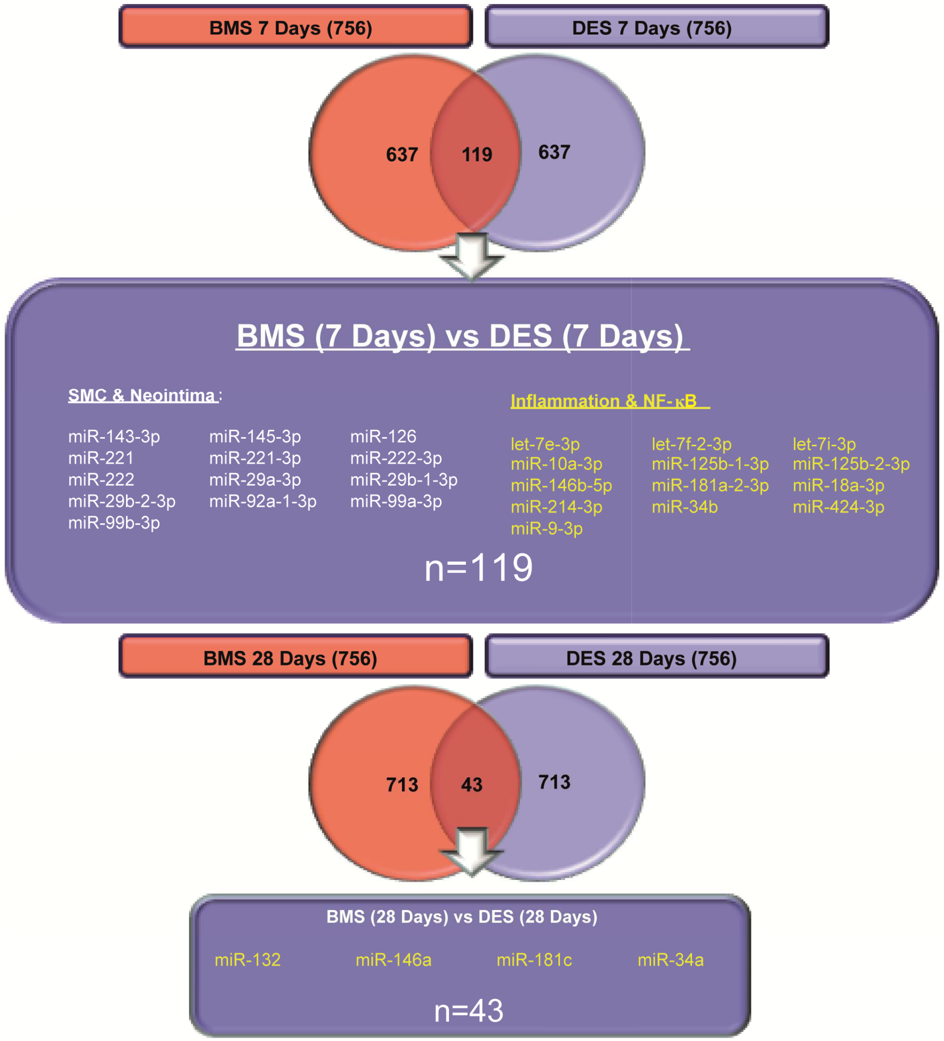

# Supplementary Figure 2

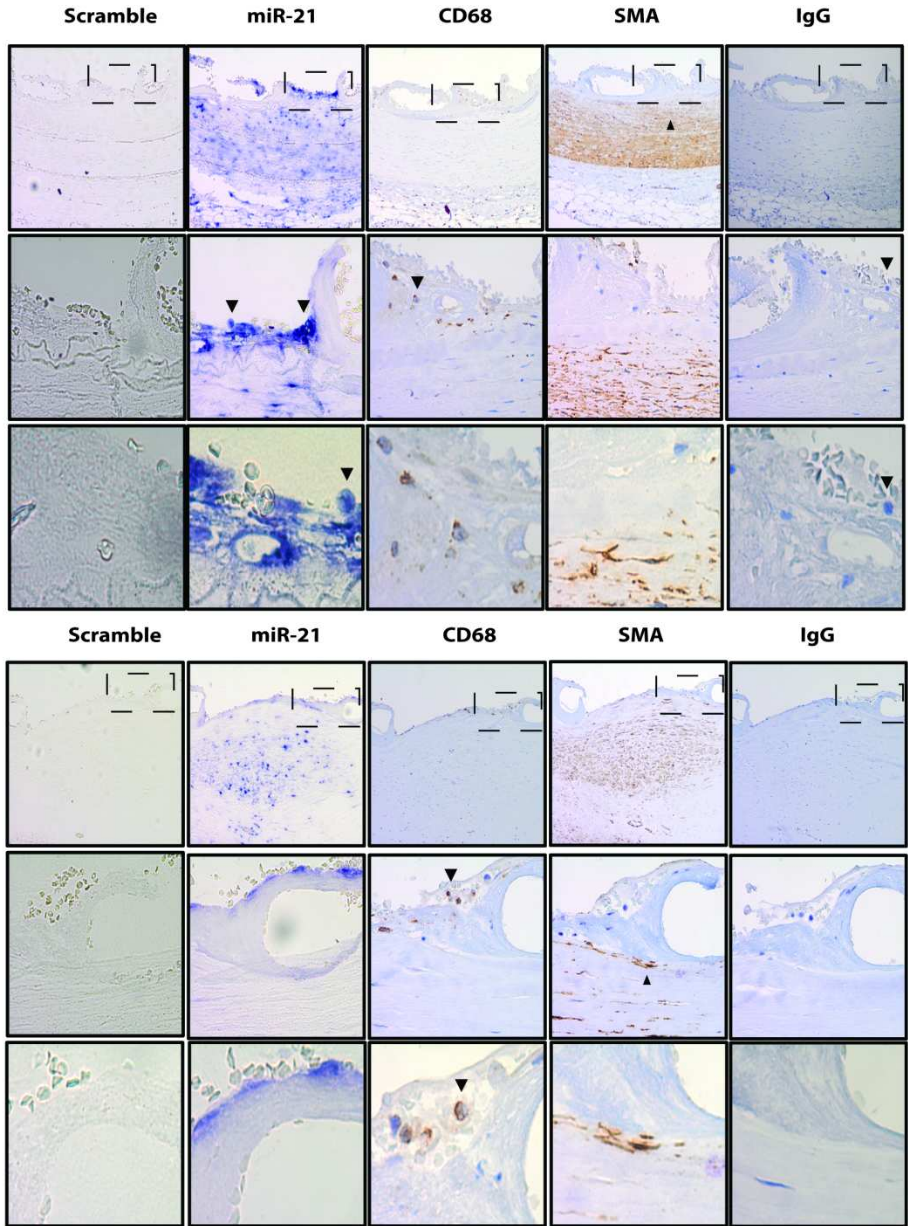

Supplementary Figure 3

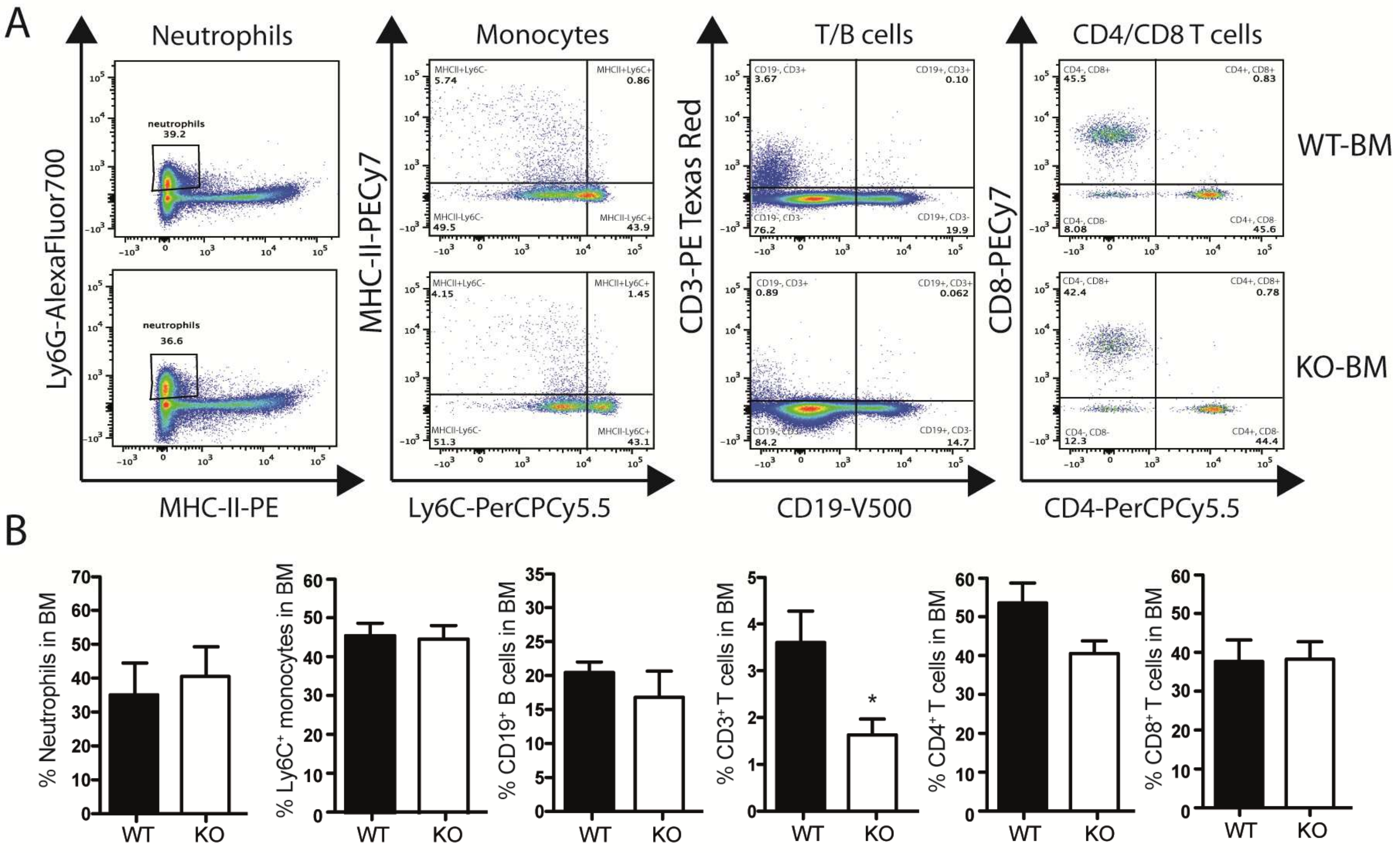

# Supplementary Figure 4

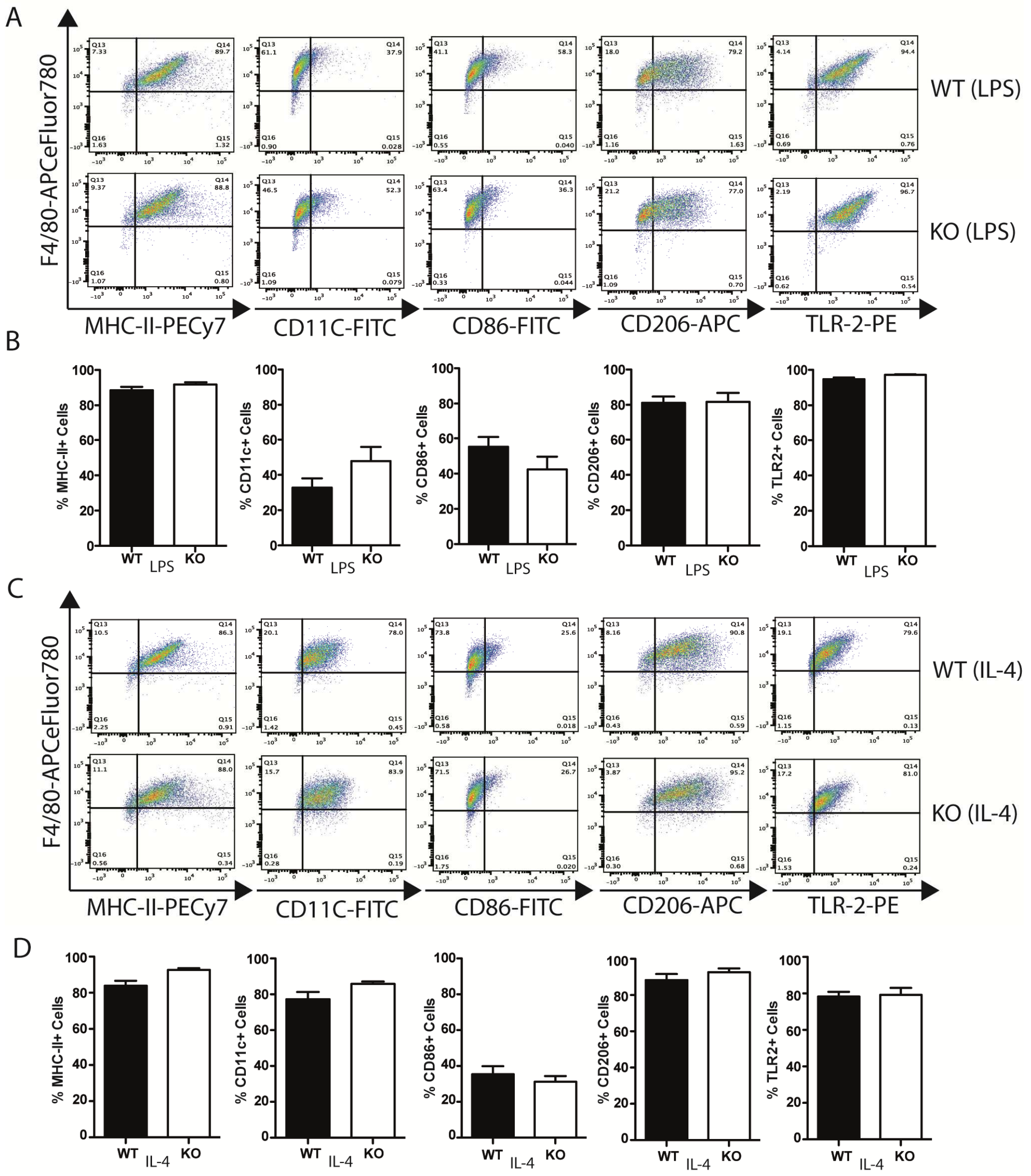

# Supplementary Figure 5

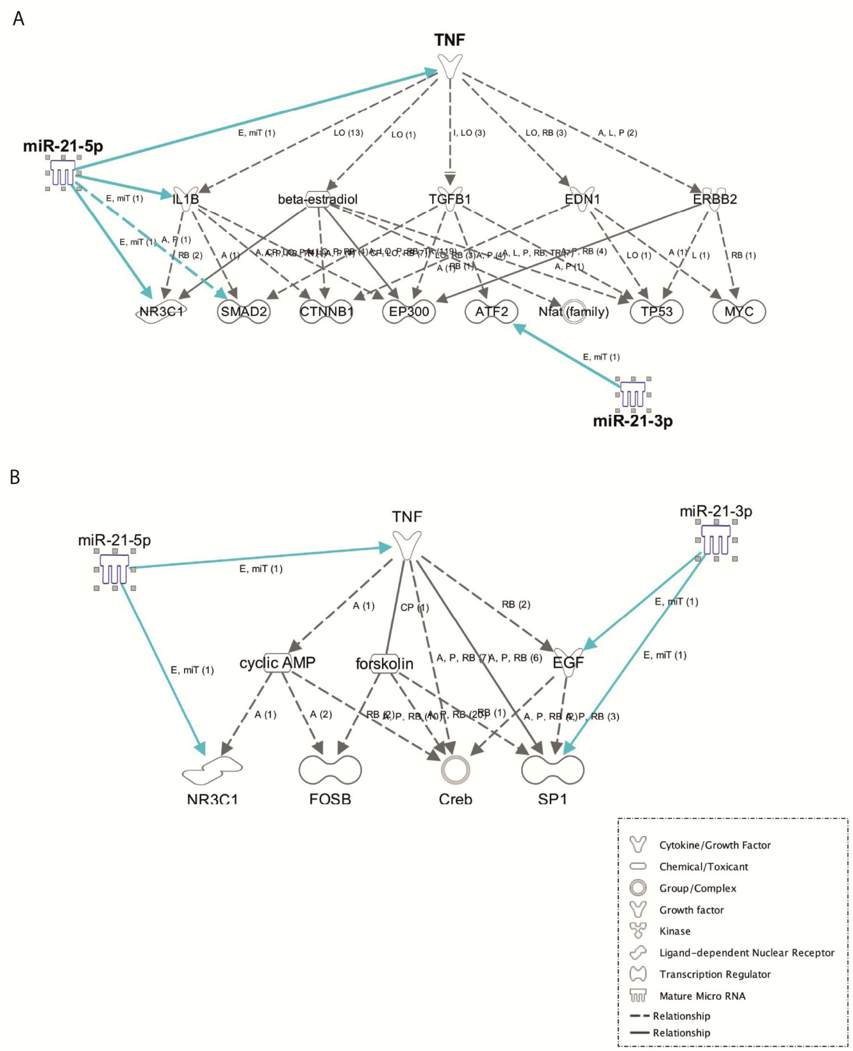

Supplement: Online Data [file mmc1.pdf]
